# MiR-206-mediated dynamic mechanism of the mammalian circadian clock

**DOI:** 10.1186/1752-0509-5-141

**Published:** 2011-09-09

**Authors:** Wei Zhou, Yan Li, Xia Wang, Lianqi Wu, Yonghua Wang

**Affiliations:** 1 Bioinformatics Center, College of Life Science, Northwest A&F University, Yangling, Shaanxi, 712100, China; 2 Department of Materials Science & Chemical Engineering, Dalian University of Technology, Dalian, Liaoning, 116023, China

## Abstract

**Background:**

As a group of highly conserved small non-coding RNAs with a length of 21~23 nucleotides, microRNAs (miRNAs) regulate the gene expression post-transcriptionally by base pairing with the partial or full complementary sequences in target mRNAs, thus resulting in the repression of mRNA translation and the acceleration of mRNA degradation. Recent work has revealed that miRNAs are essential for the development and functioning of the skeletal muscles where they are. In particular, miR-206 has not only been identified as the only miRNA expressed in skeletal muscles, but also exhibited crucial roles in regulation of the muscle development. Although miRNAs are known to regulate various biological processes ranging from development to cancer, much less is known about their role in the dynamic regulation of the mammalian circadian clock.

**Results:**

A detailed dynamic model of miR-206-mediated mammalian circadian clock system was developed presently by using Hill-type terms, Michaelis-Menten type and mass action kinetics. Based on a system-theoretic approach, the model accurately predicts both the periodicity and the entrainment of the circadian clock. It also explores the dynamics properties of the oscillations mediated by miR-206 by means of sensitivity analysis and alterations of parameters. Our results show that miR-206 is an important regulator of the circadian clock in skeletal muscle, and thus by study of miR-206 the main features of its mediation on the clock may be captured. Simulations of these processes display that the amplitude and frequency of the oscillation can be significantly altered through the miR-206-mediated control.

**Conclusions:**

MiR-206 has a profound effect on the dynamic mechanism of the mammalian circadian clock, both by control of the amplitude and control or alteration of the frequency to affect the level of the gene expression and to interfere with the temporal sequence of the gene production or delivery. This undoubtedly uncovers a new mechanism for regulation of the circadian clock at a post-transcriptional level and provides important insights into the normal development as well as the pathological conditions of skeletal muscles, such as the aging, chronic disease and cancer.

## Background

Most organisms use circadian rhythms to keep the temporal order and anticipate daily variations in their environmental changes [1]. Circadian rhythms are the sustained oscillations occurring with a periodicity close to 24 h in almost all living organisms from the cyanobacteria to plants, insects and mammals which are innately generated by an internal timing mechanism, i.e., the so-called circadian clock. In mammals, the master circadian clock is located in the suprachiasmatic nucleus (SCN) of the ventral hypothalamus [2], where it orchestrates the diurnal changes in both the physiology and behavior. As such, the SCN is capable of generating self-sustained rhythmicity in its intrinsic biological processes. The mammalian circadian clock regulates many bodily functions, such as the sleep-wake cycles, the neuroendocrine levels, mental alertness, physical strength, renal and liver activity, body temperature, blood pressure, blood viscosity and the release of appropriate hormones at different times [3]. In addition to these widely well-known effects, circadian rhythms also play a role in the pathogenesis and guide the optimal treatment for certain diseases including the arthritis, asthma, cancer, cardiovascular disease, diabetes, duodenal ulcers, hypercholesterolemia, and seasonal affective disorders [4,5].

At the molecular level, molecular and genetic studies indicate that a circadian period arises from a system of interconnected feedback loops which control the transcription of a small number of "clock" genes [6,7]. It is intriguing that the mechanism of circadian rhythms relies exactly on the interaction of these negative or positive feedback loops, which has been proposed to be important for generation of the basic circadian rhythm. In other words, every oscillator has both positive and negative elements to establish the feedback loop. The positive elements of the loop activate the expression of the negative elements, in which way moving a system away from its equilibrium state and become more unstable. Whereas, the negative elements feed back to block their own activation (as induced by the positive elements), which usually makes a system go into some equilibrium state to become more stable. Importantly, the key aspects of these feedback loops (including the period and amplitude) can be modulated by the changes in clock. Although the sustained circadian rhythms are produced by the central pacemaker located in the SCN of the anterior hypothalamus [8,9], peripheral tissues such as liver, kidney or skeletal muscle can also give rise to circadian rhythms [10]. Recent work also showed that the clock timing and entrainment could be affected at a post-transcriptional level [11]. Thus, it is important to investigate the molecular basis of the post-transcriptional, i.e., miRNA-mediated regulation of the circadian timing both in the SCN and peripheral tissues.

MiRNAs are small non-coding RNAs, with approximately 22 nucleotides long that are involved in post-transcriptional regulation of the gene expressions in most living organisms. They are well conserved in eukaryotic organisms and are thought to be vital and evolutionarily ancient components of the genetic regulation [12]. MiRNA molecules are partially complementary to one or more mRNA molecules, with a main function of down-regulating the gene expression in a variety of manners. They play a crucial role in diverse biological processes during the normal development such as the developmental timing and patterning, apoptosis, cell proliferation, organ development, as well as the pathological responses like the tumorigenesis [13,14]. However, up to now for most miRNAs, very little is known regarding their functions and precise regulation mechanisms. Thus, great efforts have been made to discover novel miRNAs and to elucidate their regulatory mechanism in recent years.

Lately, a large fraction of miRNAs were found exhibiting strict developmental stage and tissue-dependent expression manner which is critical for their appropriate activities [15], and the clock-relevant miRNAs are the same cases. Sempere et al. proved that the canonical myomiRs (myo = muscle + miR = miRNA) miR-1, -133a and -206 are highly expressed in both human and mouse heart and skeletal muscles [16]. And miR-206 is, especially, specifically restricted to the skeletal muscle, but is absent or expressed at relatively low levels in other tissues, which is exactly the reason making itself a unique one among the myomiR family [17-19]. MiR-206 reinforces the muscle differentiation program by reducing the levels of DNA polymerase and the inhibitory HLH protein Id, which functions as a negative regulator of MyoD [20]. In addition, chromatin immunoprecipitation experiments also demonstrated that the expression of muscle-enriched miRNAs was controlled by the myogenic regulatory factors Myod1 and myogenin [21], two key components of the core circadian clock. Indeed, miR-206 was recently found to be a direct transcriptional target of Myod1 [22], a muscle-specific regulator that can stimulate the expression of miR-206. Despite of the role as a clock-controlled gene, Myod1 was also a constituent of the skeletal muscle circadian transcriptome [23]. Up to now, the Clock gene is the only reported miR-206 target involved in the circadian mechanism [24]. Taken together, the unique exclusively and highly expressed muscle-specific miRNA-206 might represent an intriguing tool to regulate the circadian rhythm process [25].

It is known that circadian clock system involves many components and complex interactions in living organisms [26]. Clearly, this complexity renders it extremely difficult to intuitively understand the clock control mechanism or even a simple configuration of those core genes. To explore the specific effects of various genes that control the circadian clocks, it is necessary to resort to computational systems biology methods, which have emerged as powerful tools to supplement the experimental work and provide insights into the operation of the gene networks. Traditionally, the modeling of chemical reactions can be achieved either by using differential equations built based on the law of mass action or by the use of their stochastic counterpart [27,28]. The modeling of circadian rhythms has also begun a long time before as recently reviewed [29,30]. As a result, many interesting properties of the rhythms have been uncovered by the mathematical models, like the discovery that there might exist multiple sources of periodic behavior in the genetic regulatory network controlling the circadian oscillations. Recently a new deterministic model for study of the mammalian circadian clock has been proposed [31]. This model was developed based on incorporation of the intertwined positive and negative regulatory loops involving the Per, Cry, Bmal1, Clock and Rev-Erbα genes, which account for the autonomous, sustained circadian oscillations in conditions corresponding to continuous darkness. Interestingly, the importance of the miRNA regulation in circadian oscillators has also been demonstrated in two dynamics models recently reported [32].

However, up to now, little is known about the contribution effects of the translational controls to the circadian rhythms system. No further model for the mammalian circadian clock involved in the miRNA-mediated regulation mechanism has been developed, particularly for the miR-206, a critical fine-tuner of the core circadian clock. To address this issue and in particular to explore the translational control by miRNAs, in the present work we attempt to establish a detailed quantitative mathematical model for the circadian rhythms oscillators in mammalian clock, which involves a Clock-Myod1-miR-206 feedback loop controlling the timing of the circadian cycle. The process includes several aspects. Firstly, the generation of the basic circadian rhythm in our model depends on two negative autoregulations of the gene expression. One is the negative autoregulatory feedback exerted by the CLOCK-BMAL1 on the expression of Bmal1 gene. The other is the Clock expression which is subjected to a negative autoregulation by the CLOCK protein. Secondly, besides the negative autoregulatory feedback of the gene expression, the interplay of the negative and positive feedback loops is also taken into consideration. Actually, these interlocked feedback loops potentially allow for multiple inputs and outputs at different phases [33] and help to dissect their differential roles in the system. As a result, a theoretical model was developed in this work and the dynamic mechanism it uncovered is well supported by several recent experimental studies [34-37]. The model and related discovery might be helpful for deep understanding of the mechanism of miRNA-mediated circadian rhythms. It points out the crucial role of the miRNA-mediated control on the gene expression during the skeletal muscle development and disease, and considers the potential of miRNAs as therapeutic targets.

## Results and discussion

### Dynamics of the pathway

Recent studies showed that the molecular mechanism of circadian oscillations not only relies on the feedback loops of the gene expression, but also is affected by the interfering of miRNAs in the circadian rhythm choreography [11]. Therefore, in this work, a miR-206-mediated mammalian circadian clock model was developed and the properties of the oscillations involved in the model were investigated which incorporated the main components that play roles in the circadian rhythms.

Using a set of parameters with appropriate biological values, the modeling work was carried out presently (Table 1) by numerical integration of Eqs. (1)-(19). The reasonability of this model can be demonstrated by the simulated periods of oscillations which are close to 24 h in continuous darkness for those key components like CLOCK protein. These oscillations are constant and self-sustained ("built-in") since the conditions of the equations are constant in time. However, because some parameter values remain to be determined experimentally, the occurrence of autonomous sustained oscillations accounted by the model might suffer from a semiarbitrary choice of parameter values. Despite of this, the model we developed still yields a period of oscillations in continuous darkness close to 24 h, as all these parameters are within a reasonable physiological range (Table 1). The observed dynamics of the clock system are well consistent with the general principle that the circadian rhythms in mammals can persist in continuous darkness or light [38].

**Table 1:** Basal parameter values yielding circadian oscillations in conditions corresponding to continuous darkness.

Parameter	Definition	Set1	Set2	Set3	Set4	Determination
*k*_1_(h^-1^)	Rate constant for entry of the BMAL1 protein into the nucleus	0.4	0.4	0.4	0.4	38
*k*_2_(h^-1^)	Rate constant for exit of the BMAL1 protein from the nucleus	0.2	0.2	0.2	0.2	38
*k*_3_(nM^-1^·h^-1^)	Rate constant for the formation of the inactive I_N _complex	0.55	0.55	0.55	0.55	38
*k*_4_(h^-1^)	Rate constant for the dissociation of the I_N _complex	0.1	0.1	0.1	0.1	38
*k*_5_(h^-1^)	Rate constant for entry of the CLOCK protein into the nucleus	2.0	2.0	2.0	2.0	24
*k*_6_(h^-1^)	Rate constant for exit of the CLOCK protein from the nucleus	1.0	1.0	1.0	1.0	24
*d*_1_(nM·h^-1^)	Rate constant for the production of the Bmal1	3.6	3.6	3.6	3.6	38, 43
*d*_2_(h^-1^)	Maximum rate of Bmal1 degradation	0.9	0.9	0.9	0.9	38, 43
*d*_3_(nM·h^-1^)	Rate constant for the production of the Myod1	4.0	4.0	4.0	4.0	38, 43
*d*_4_(h^-1^)	Maximum rate of Myod1 degradation	0.9	0.9	0.9	0.9	38, 43
*d*_5_(nM·h^-1^)	Rate constant for the production of the Clock	4.0	4.0	4.0	4.0	38, 43
*d*_6_(h^-1^)	Maximum rate of Clock degradation	0.9	0.9	0.9	0.9	38, 43
*K_IB_*(nM)	Inhibition constant for repression of Bmal1 expression by nuclear BMAL1	0.8	0.8	0.8	0.8	38
*K_I_*(nM)	Inhibition constant for repression of Clock expression by nuclear CLOCK	2.0	2.0	2.0	2.0	24
*K_AM_*(nM)	Activation constant for enhancement of miR-206 expression by MYOD1	2.0	2.0	2.0	2.0	24, 38
*K_AC_*(nM)	Activation constant for enhancement of Myod1 expression by the I_N _complex	0.8	0.8	0.8	0.8	38
*k_dmb_*(h^-1^)	Nonspecific degradation rate constant for Bmal1 mRNA	0.01	0.01	0.01	0.01	38
*k_dmc_*(h^-1^)	Nonspecific degradation rate constant for Myod1 mRNA	0.12	0.12	0.12	0.12	38
*k_dnc_*(h^-1^)	Nonspecific degradation rate constant for cytosolic non-phosphorylated MYOD1	0.5	0.5	0.5	0.5	38
*k*_*dn*1_(h^-1^)	Nonspecific degradation rate constant for cytosolic dephosphorylated BMAL1	0.01	0.01	0.01	0.01	38
*k*_*dn*2_(h^-1^)	Nonspecific degradation rate constant for cytosolic phosphorylated BMAL1	0.01	0.01	0.01	0.01	38
*k*_*dn*3_(h^-1^)	Nonspecific degradation rate constant for nuclear dephosphorylated BMAL1	0.01	0.01	0.01	0.01	38
*k*_*dn*4_(h^-1^)	Nonspecific degradation rate constant for cytosolic phosphorylated MYOD1	0.01	0.01	0.01	0.01	38
*k*_*dn*5_(h^-1^)	Nonspecific degradation rate constant for I_N _complex	0.01	0.01	0.01	0.01	38
*k*_*dn*6_(h^-1^)	Nonspecific degradation rate constant for nuclear phosphorylated BMAL1	0.01	0.01	0.01	0.01	38
*K*_*d*1_(nM)	Michaelis constant for cytosolic phosphorylated BMAL1 degradation	0.3	0.3	0.3	0.3	38
*K*_*d*2_(nM)	Michaelis constant for nuclear phosphorylated BMAL1 degradation	0.3	0.3	0.3	0.3	38
*K*_*d*3_(nM)	Michaelis constant for cytosolic phosphorylated MYOD1 degradation	0.3	0.3	0.3	0.3	38
*K*_*d*4_(nM)	Michaelis constant for I_N _complex degradation	0.3	0.3	0.3	0.3	38
*K*_*dp*1_(nM)	Michaelis constant for cytosolic BMAL1 dephosphorylation	0.3	0.3	0.3	0.3	38
*K*_*dp*2_(nM)	Michaelis constant for nuclear BMAL1 dephosphorylation	0.3	0.3	0.3	0.3	38
*K*_*dp*3_(nM)	Michaelis constant for cytosolic MYOD1 dephosphorylation	0.3	0.3	0.3	0.3	38
*K*_*p*1_(nM)	Michaelis constant for cytosolic BMAL1 phosphorylation	0.1	0.1	0.1	0.1	38
*K*_*p*2_(nM)	Michaelis constant for nuclear BMAL1 phosphorylation	0.1	0.1	0.1	0.1	38
*K*_*p*3_(nM)	Michaelis constant for cytosolic MYOD1 phosphorylation	0.1	0.1	0.1	0.1	38
*K_vd_*(nM)	Michaelis constant for P_2 _degradation	0.1	0.1	0.1	0.1	24
*K*_1_(nM)	Michaelis constant for P_0 _phosphorylation	1.5	1.5	1.5	1.5	24
*K*_2_(nM)	Michaelis constant for P_1 _dephosphorylation	2.0	2.0	2.0	2.0	24
*K*_3_(nM)	Michaelis constant for P_1 _phosphorylation	1.5	1.5	1.5	1.5	24
*K*_4_(nM)	Michaelis constant for P_2 _dephosphorylation	2.0	2.0	2.0	2.0	24
*K_mB_*(nM)	Michaelis constant for degradation of Bmal1 mRNA	0.4	0.4	0.4	0.4	38
*K_mC_*(nM)	Michaelis constant for degradation of Myod1 mRNA	0.1	0.1	0.1	0.1	38
*K_m_*(nM)	Michaelis constant for degradation of Clock mRNA	0.2	0.2	0.2	0.2	24
*k_stot _*(h^-1^)	Rate constant for protein synthesis	1.0	1.0	1.0	1.0	38
*k_sB_*(h^-1^)	Rate constant for synthesis of BMAL1	1.0k_stot_	1.0k_stot_	1.0k_stot_	1.0k_stot_	38
*k_sC_*(h^-1^)	Rate constant for synthesis of MYOD1	0.3k_stot_	0.3k_stot_	0.3k_stot_	0.3k_stot_	38
*k_s_*(h^-1^)	Rate constant for synthesis of CLOCK	2.0	2.0	2.0	2.0	24
*m*_1_	Degree of cooperativity of repression of Bmal1 expression by nuclear dephosphorylation BMAL1	4.0	4.0	4.0	4.0	38
*m*_2_	Degree of cooperativity of repression of Clock expression by nuclear CLOCK	4.0	4.0	4.0	4.0	38
*n*_1_	Degree of cooperativity of activation of Myod1 expression by I_N _complex	4.0	4.0	4.0	4.0	38
*n*_2_	Degree of cooperativity of activation of miR-206 synthesis by cytosolic dephosphorylation MYOD1	4.0	4.0	4.0	4.0	38
*V*_1*B*_(nM·h^-1^)	Maximum rate of cytosolic BMAL1 phosphorylation	1.0	1.0	1.0	1.0	38
*V*_2*B*_(nM·h^-1^)	Maximum rate of cytosolic BMAL1 dephosphorylation	0.1	0.1	0.1	0.1	38
V_3*B*_(nM·h^-1^)	Maximum rate of nuclear BMAL1 phosphorylation	1.0	1.0	1.0	1.0	38
*V*_4*B*_(nM·h^-1^)	Maximum rate of nuclear BMAL1 dephosphorylation	0.2	0.2	0.2	0.2	38
*V*_1*c*_(nM·h^-1^)	Maximum rate of cytosolic MYOD1 phosphorylation	1.0	1.0	1.0	1.0	38
*V*_2*c*_(nM·h^-1^)	Maximum rate of cytosolic MYOD1 dephosphorylation	0.5	0.5	0.5	0.5	38
*V*_1_(nM·h^-1^)	Maximum rate of cytosolic P_0 _phosphorylation	6.0	6.0	6.0	6.0	24
*V*_2_(nM·h^-1^)	Maximum rate of cytosolic P_1 _dephosphorylation	3.0	3.0	3.0	3.0	24
*V*_3_(nM·h^-1^)	Maximum rate of cytosolic P_1 _phosphorylation	6.0	6.0	6.0	6.0	24
*V*_4_(nM·h^-1^)	Maximum rate of cytosolic P_2 _dephosphorylation	3.0	3.0	3.0	3.0	24
*V_dBC_*(Nm·h^-1^)	Maximum rate of degradation of cytosolic phosphorylated BMAL1	1.0	1.0	1.0	1.0	38
*V_dBN_*(nM·h^-1^)	Maximum rate of degradation of nuclear phosphorylated BMAL1	1.0	1.0	1.0	1.0	38
*V_dcc_*(nM·h^-1^)	Maximum rate of degradation of cytosolic phosphorylated MYOD1	0.1	0.1	0.1	0.1	38
*V_dIN_*(nM·h^-1^)	Maximum rate of degradation of I_N _complex	0.6	0.6	0.6	0.6	38
*V_mB_*(nM·h^-1^)	Maximum rate of Bmal1 mRNA degradation	0.3	0.3	0.3	0.3	38
*V_mC_*(nM·h^-1^)	Maximum rate of Myod1 mRNA degradation	0.12	0.12	0.12	0.12	38
*V_d_*(nM·h^-1^)	Maximum rate of P_2 _degradation	1.5	1.5	1.5	1.5	24
*v_stot_*(nM·h^-1^)	Maximum transcription rate	1.0	1.0	1.0	1.0	38
*V_sB_*(nM·h^-1^)	Maximum rate of Bmal1 mRNA synthesis	0.28v_stot_	0.28v_stot_	0.28v_stot_	0.28v_stot_	38
*V_sC_*(nM·h^-1^)	Maximum rate of Myod1 mRNA synthesis	0.95v_stot_	0.95v_stot_	0.95v_stot_	0.95v_stot_	38
*V_sM_*(nM·h^-1^)	Maximum rate of miR-206 synthesis	1.0	1.0	1.0	1.0	24, 38
*V_s_*(nM·h^-1^)	Maximum rate of Clock mRNA synthesis	0.5	0.5	0.5	0.5	24
*V_m_*(nM·h^-1^)	Maximum rate of Clock mRNA degradation	0.3	0.3	0.3	0.3	24
*C*_1_(nM·h^-1^)	Rate constant for the production of the miR-206	0.01	0.01	0.01	0.01	43
*C*_2_(h^-1^)	Maximum rate of miR-206 degradation	0.01	0.01	0.01	0.01	43
*C*_3_(nM^-1^·h^-1^)	Rate constant for the formation of the R_ISC _complex	0	0.5	1.0	1.5	43
*C*_4_(h^-1^)	Maximum rate of R_ISC _complex degradation	0.6	0.6	0.6	0.6	43

The sets 1, 2, 3 and 4 represent the cases for the *R_ISC _*formation rate *C*_3 _= 0, 0.5, 1.0 and 1.5 nM^-1^·h^-1^, respectively. The oscillation periods of the four parameter sets are 24.6, 23.8, 24 and 24.04 h respectively. The parameter set 3 was selected because the circadian oscillation period is consistent with the physiologic phenomenon.

**Figure 1:**
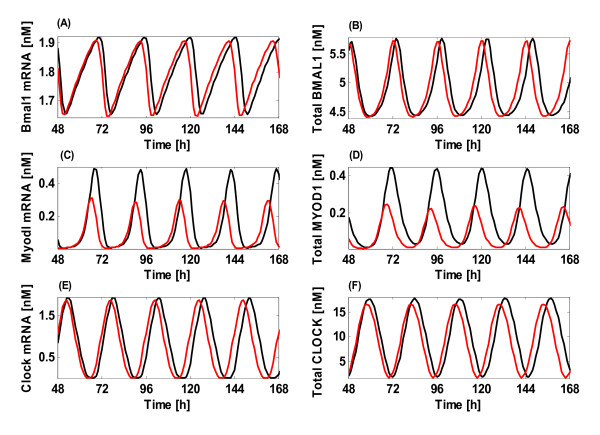
**Circadian oscillations in constant darkness**. (A, C and E) Time evolution of the mRNAs of Bmal1 (*M_B_*), Myod1 (*M_my_*) and Clock (*M_P_*). (B, D and F) Corresponding oscillations of the total amounts of BMAL1, MYOD1 and CLOCK proteins. The red and black curves show the oscillations with or without the regulation of miR-206, respectively.

Figure 1 shows the obtained results where the red curves represent the oscillations with the regulation of miR-206. With introduction of miR-206, we can see that the oscillations of Bmal1 mRNA (*M_B_*), Myod1 mRNA (*M_my_*) and Clock mRNA (*M_P_*) are all kept with a period of ~24 h (Figures 1A, C and 1E). The proteins follow their mRNAs by a few hours which also go through similar oscillations (Figures 1B, D and 1F). In addition, the Bmal1 mRNA oscillates in phase with Myod1 mRNA, but in antiphase with Clock mRNA. These results are in a good agreement with those experimental observations [39,40], proving the reasonability of the model. As we know, circadian rhythm in mammals can generate sustained oscillations that are mainly produced by the SCN, a pair of distinct groups of cells located in the hypothalamus [8,9]. Other peripheral mammalian tissues such as liver, heart, kidney and skeletal muscle [10] can also generate circadian rhythms, leading to endogenous and continuous rhythmic oscillations in aperiodic physiological response. Besides, presently miR-206 was also demonstrated to be a potentially novel avenue by which the circadian timing processes in skeletal muscle are regulated.

The black curves of Figure 1 representing those oscillations of the system in the absence of miR-206 disclose some other interesting information. As seen from the curves, obviously without the miR-206 regulation only a subtle change is observed in the oscillatory pattern of the system compared to the miR-206-mediated case, indicating that the impact of miRNA regulation on the oscillatory pattern formation in circadian rhythms may not be enormous. However, the oscillatory appearance of some key components such as Myod1 mRNA and its protein changes significantly, with the amplitude increased by about 77% and 133% (Figures 1C and 1D) respectively, but the increase in the amplitude of Bmal1 mRNA, Clock mRNA and their proteins is less pronounced as approximately 10% (Figures 1A, B, E and 1F).

**Figure 2:**
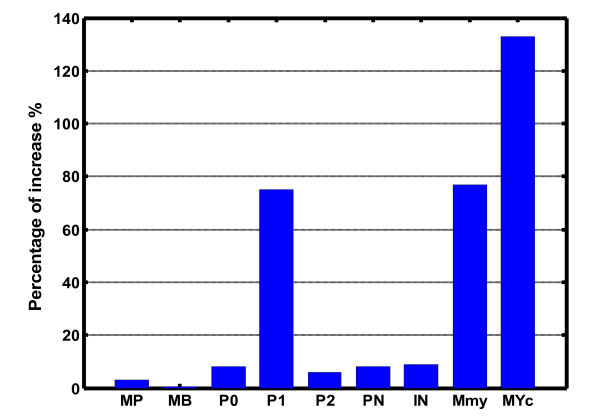
**The expression proportion of key components in the pathways with miR-206 mediation to that without miR-206 mediation**.

The same tendency observed in Figure 1 is also proven by Figure 2, which accurately depicts the expression increase for those key components in the pathways with or without the miR-206 regulations. Clearly, for several crucial components like the Clock mRNA (*M_P_*), Bmal1 mRNA (*M_B_*), cytosolic and nuclear CLOCK proteins (*P*_0_, *P*_2_, *P_N_*) and the complex between the nuclear CLOCK and BMAL1 proteins (*I_N_*), only small variations in the amplitude of the components are observed without the miR-206 regulation. But for other components, like the cytosolic CLOCK proteins (*P_1_*), Myod1 mRNA (*M_my_*) and cytosolic MYOD1 (*MY_C_*), their expression levels are largely enhanced in the absence of miRNA. This may be due to the fact that MyoD is a direct target of the circadian transcriptional activators *I_N_*, which binds in a rhythmic manner to the core enhancer of the MyoD promoter [41]. Therefore, the complex *I_N _*directly controls the *M_my _*and *MY_C _*expression, which is more important than the indirect miRNA regulation. These results may be related to the fact that the transcription and translation of key circadian core components, on one hand are tightly connected with each other, and on the other hand may buffer each other. Such a regulatory feature could also explain why a major increase in the transcription, like the one caused by the myod1 gene, results in only a modest increase in Myod1 mRNA abundance and probably an even more modest increase in the translated protein.

From the above results, three interesting findings are presented: 1) Although the miR-206 regulation is crucial in controlling the dynamics of the circadian rhythms system, it is not strong and fits exactly some recent data demonstrating that a Drosophila miRNA can function as a buffering agent against the environmental perturbations during the development [42]. 2) Sustained oscillations with a circadian period close to 24 h occur in our model in the continuous darkness, which is precisely regulated by the interlocked positive and negative feedback loops. And these loops effectively maintain the oscillations and the stability of the system, as indicated by the small variation in the amplitude of several key components (such as the *M_P_*, *M_B_*, *P_0_*, *P_2_*, *P_N _*and *I_N_*). But the expression levels of cytosolic CLOCK proteins, Myod1 mRNA and cytosolic MYOD1 are largely enhanced in the absence of miRNA, reflecting the fact that the complex *I_N_*'s direct effects on the *M_my _*and *MY_C _*expression is more important than the indirect miRNA regulation. These observations may provide a plausible mechanism through which tissue-specific factors such as Myod1 and miR-206 can convey unique tissue requirements to the circadian clock. 3) Once again, it is confirmed that miR-206 is necessary for accurate circadian timekeeping, since in the absence of miRNA, the oscillation period of the system as shown by the three representative components, Bmal1, Myod1 and Clock mRNAs and their corresponding proteins is not very constant and changes with time. At 48 h, the period is 24.5 h. But when the simulation time increases to 300 h, the period has changed up to ~24.6 h, and even up to ~24.7 h at 1000 h or much longer time. However the system is more stable in the presence of miRNA when the period of the oscillatory system is always 24.0 h, which further indicates the important role of miR-206 plays in the accurate circadian timekeeping.

### Dynamic sensitivity analysis

**Figure 3:**
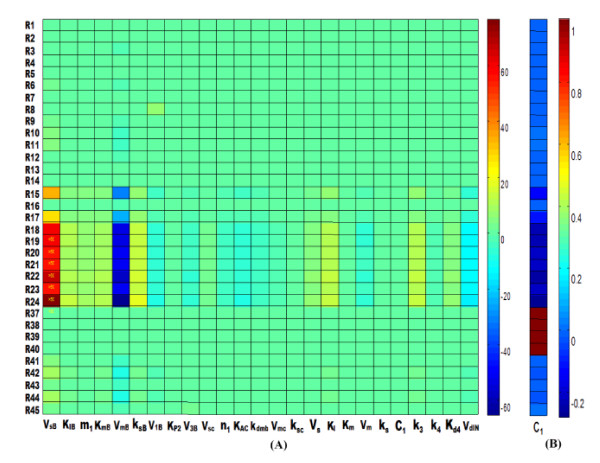
**The heat maps of local sensitivities of each reaction flux with respect to each parameter**. Each column represents a parameter, and each row represents a single reaction flux. Reactions R_1_-R_24 _represent the Bmal1 mRNA formation, Bmal1 mRNA degradation, cytosolic BMAL1 formation, cytosolic BMAL1 phosphorylation, cytosolic BMAL1 dephosphorylation, cytosolic BMAL1 nonspecific degradation, cytosolic phosphorylated BMAL1 degradation, cytosolic phosphorylated BMAL1 nonspecific degradation, BMAL1 protein entry into the nucleus, BMAL1 protein exit from the nucleus, nuclear BMAL1 nonspecific degradation, nuclear BMAL1 phosphorylation, nuclear BMAL1 dephosphorylation, nuclear phosphorylated BMAL1 degradation, Myod1 mRNA formation, Bmal1 mRNA nonspecific degradation, Myod1 mRNA degradation, Myod1 mRNA nonspecific degradation, MYOD1 formation, cytosolic MYOD1 phosphorylation, cytosolic MYOD1 dephosphorylation, cytosolic MYOD1 degradation, cytosolic phosphorylated MYOD1 degradation, cytosolic phosphorylated MYOD1 nonspecific degradation, respectively. Reactions R_37_-R_45 _represent the miR-206 formation, *R_ISC _*complex formation, miR-206 degradation, *R_ISC _*complex degradation, *I_N _*complex formation, *I_N _*complex dissociation, *I_N _*complex degradation, *I_N _*complex nonspecific degradation and nuclear phosphorylated BMAL1 nonspecific degradation, respectively. The explanation of the parameters in each column can be seen in Table 1.

Having many successfully applications in the study of biochemical systems [27,43], sensitivity analysis provides a systematic framework to investigate how changes of a parameter cause changes of the dynamic behavior of a pathway, and which parameters are the most crucial ones impacting the whole system. In the present work, a sensitivity analysis for all parameters (Table 1) of the mammalian circadian pathway mediated by miR-206 was conducted, when a total of 3724 (76 rate constants × 49 reactions) local sensitivities were calculated and normalized with 288 scaled sensitivity absolute values (|S|). As a result, 47 rate constants are found larger than 0.9, where negative S indicates that the reaction output decreases with the increasing rate constant. In Figure 3A, only those reactions or parameters with significant effects as revealed by the sensitivity analysis on the system are shown, and others with weak or no influences (|S| < 1) on the pathway were omitted for clarity.

The rate of Bmal1 mRNA synthesis (*V_sB_*) has the largest impact on the whole system, affecting 21 out of the total 49 reactions while *V_mB _*significantly affects 20 reactions, *K_sB _*affects 14 reactions, and *V_1B_, V_3B _*and *K_I _*respectively affecting 13 reactions (Figure 3). These observations indicate that the impact of these parameters on the whole pathway are in the order of *V_sB _*>*V_mB _*>*K_sB _*>*V_1B _*>*V_3B _*>*K_I_*. Thus, we may conclude that the parameters with respect to the synthesis and degradation of BMAL1 and its mRNA exert a marked effect on producing sustained oscillations. In other words, the clock mechanism is highly sensitive to BMAL1. This might be a result of the critical role of BMAL1 promoting the circadian periodicity of the transcriptional activation, since it takes shape a positive branch of the main transcriptional autoregulatory feedback loop.

In addition, another interesting finding is that parameter *C*_1 _which relates to the production of miR-206 affects 4 reactions of all 49 reactions with |S| = 1. This positively shows the importance of miR-206 in regulating the whole system. Figure 3B depicts this result by the red region that represents the influence of *C*_1 _on the model. The small value of the sensitivity indices (|S| = 1) reflects a fact that miRNA though regulating the system in a relatively weak manner, is still crucial in controlling the dynamics of the circadian clock.

**Figure 4:**
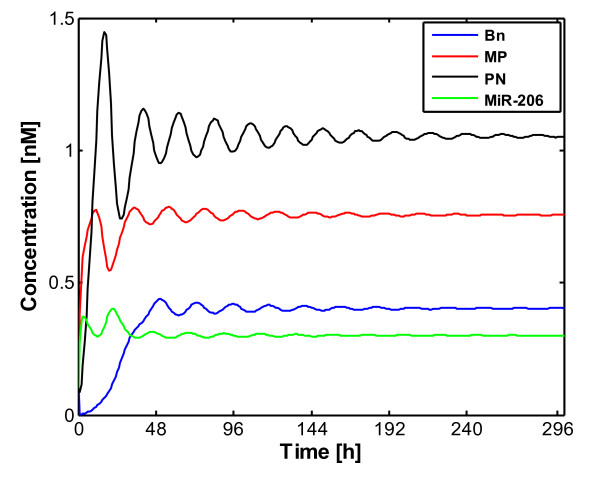
**Damped oscillations that occur in the model in continuous darkness**. The curves showing the time evolution of *B_n_*, *M_P_*, *P_N _*and MiR-206 were obtained when *C*_1 _= 0.23 nM·h^-1 ^while other parameters kept the same basal values as used in Figure 1.

As we know, sustained oscillations only occur within an appropriate range of parameter values, and beyond this range the system tends to reach a steady state. Such an evolution is often associated with the occurrence of damped oscillations. The mammalian circadian system is thought to be composed of a hierarchical set of oscillators with the suprachiasmatic nuclei acting as a master pacemaker [2,44] which is independently able to both generate and sustain its own circadian oscillations. The peripheral tissues can give rise to circadian rhythms and these peripheral rhythms also appear to be sustained [45]. Peripheral rhythms are damped unless they are driven by periodic signals received from the SCN [46,47]. The oscillations can readily be entrained by the periodic variation of the parameter when damped oscillations occur in the model in continuous darkness [31]. All these observations can be found in the present model in Figure 4, which displays the time evolution curves of *B_n_*, *M_P_*, *P_N _*and MiR-206. These curves were obtained when *C*_1 _= 0.23 nM·h^-1 ^and all other parameters remained as the same values as in Figure 1.

### The effect of parameter on model dynamics

**Figure 5:**
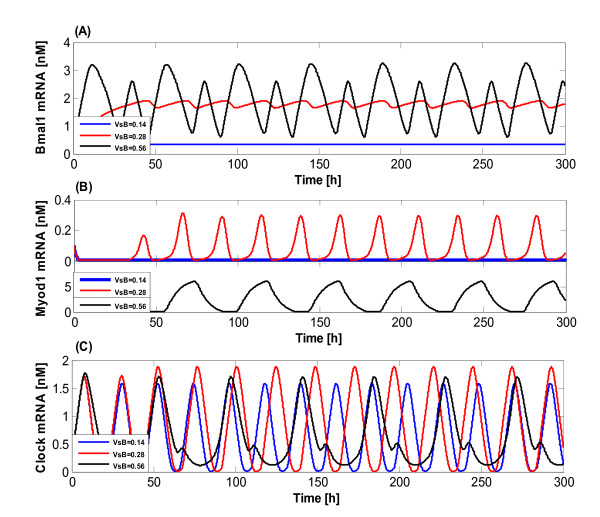
**Temporal behavior of the model with miRNA regulation**. The blue, red and black curves represent the cases for *V_sB _*= 0.14, 0.28 and 0.56 nM·h^-1^, respectively.

As a starting point to examine how the synthesis rate of Bmal1 mRNA (*V_sB_*) exhibits the largest effects on our system, some explorations on variation of the parameter space were carried out presently. The value of *V_sB _*was changed as 0.14, 0.28 (physiological value) and 0.56 nM·h^-1^, when values of other parameters were kept fixed. Figure 5 shows the obtained results where the blue, red and black curves represent the cases when *V_sB _*= 0.14, 0.28 and 0.56 nM·h^-1^, respectively. When *V_sB _*decreases from 0.28 to 0.14 nM·h^-1^, the oscillations of Bmal1 mRNA (Figure 5A) and Myod1 mRNA (Figure 5B) vanish and subsequently evolve toward a steady state. The oscillation amplitude of Clock mRNA (Figure 5C) decreases accompanied with the increase of the oscillation frequency. However, as *V_sB _*increases to 0.56 nM·h^-1^, an unequal amplitude of oscillation occurs for Bmal1 mRNA with a decreasing oscillation period compared to the 'basal' value of *V_sB _*= 0.28nM·h^-1 ^(Figure 5A). Since *V_sB _*is the synthesis rate of Bmal1 mRNA, the amplitude of oscillation increases when *V_sB _*rises to 0.56 nM·h^-1^. The reason for the occurrence of unequal amplitude of oscillation for Bmal1 mRNA might be due to the negative feedback control on the Bmal1 mRNA production from the downstream gene like *I_N _*complex (see Figure 10 in the latter part). But *V_sB _*plays an indirect role for the Myod1 mRNA production. This also explains why Myod1 mRNA oscillates in an equal amplitude and in a constant period in regard to *V_sB _*= 0.28 nM·h^-1 ^(Figure 5B), since there is no any negative feedback for the Myod1 mRNA (see Figure 10 in the latter part).

In contrast, the oscillation of Clock mRNA keeps constant with only slight variations occurring in the amplitude and period with the change of *V_sB _*(Figure 5C). This is consistent with the Clock expression dynamics to maintain normal circadian rhythmicity [48]. A more interesting finding here is that the system still generates sustained oscillation even when *V_sB _*value is whether enlarged or lessened (from 0.1 to 3.0), indicating that the Clock is robust to the variations of *V_sB_*. This on one hand might explain why the biological clock period is always constant in various organisms, and on the other hand is also consistent with the fact that the Clock expression is necessary to maintain the normal circadian rhythmicity [48]. Clearly, the robustness of the CLOCK protein would play a crucial role in a living system to keep its normal physiological functions, since the developed biological clocks from evolution in animals, plants and others are important in regulating and coordinating the internal biological processes [49].

**Figure 6:**
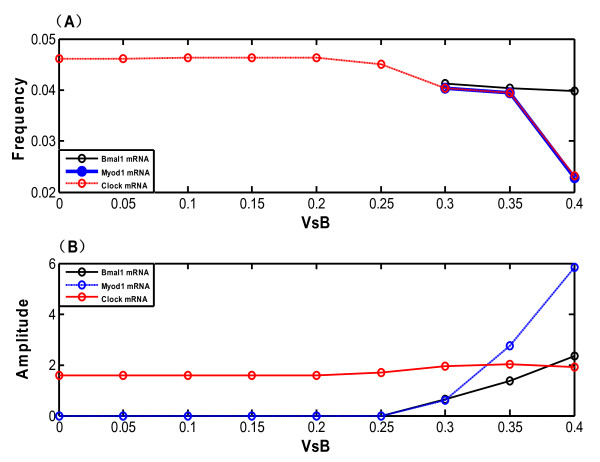
**Effect of the variation of the maximum degradation rate of Bmal1 mRNA (*V_sB _*nM·h^-1^) on the model**. Variation of (A) the frequency and (B) the amplitude of oscillation in the model as a function of *V_sB _*(nM·h^-1^). Bmal1 mRNA, Myod1 mRNA and Clock mRNA are shown in black, blue and red, respectively.

Figure 6 shows how the frequency and amplitude of the oscillations for Bmal1, Myod1 and Clock mRNAs vary with the increase of *V_sB _*value when keeping all other rate constants fixed (Figure 6). The black, blue, and red curves correspond to the Bmal1 mRNA, Myod1 mRNA and Clock mRNA, respectively. The oscillation of Bmal1 and Myod1 mRNAs disappears and the system evolves toward a stable state when *V_sB _*≤ 0.25 nM·h^1 ^(Figure 6A). In other words, the period and amplitude of the oscillation in regard to the two mRNAs equal 0, i.e., their frequency tends to infinity in this case. However, when *V_sB _*> 0.25 nM·h^-1^, the frequency of oscillation related to Bmal1 mRNA becomes stable while the frequency of oscillation related to Myod1 mRNA declines sharply. The frequency of Clock mRNA also decreases with the increase of *V_sB_*. The amplitude of oscillation for Bmal1 and Myod1 mRNAs tends to increase, while there is only a marginal change in the amplitude with regard to Clock mRNA (Figure 6B). All the above data not only show the significant roles of parameter *V_sB _*in the whole system, but also prove the robustness of the clock mechanism to system perturbations, which might be due to a direct and negative autoregulation in the dynamic system.

### The effect of parameter variation of miRNA

Previous research suggested that miR-206 is specifically expressed in skeletal muscle [50], which is a clock-controlled gene that plays a role in regulating the peripheral circadian rhythm. The sensitivity analysis here also shows that the system is sensitive to the variation of the miR-206 production rate (*C*_1_). Therefore, an attempt has been made to investigate how the changes of the kinetic parameters associated with miR-206 cause changes in the dynamic behavior of the biological clock system.

**Figure 7:**
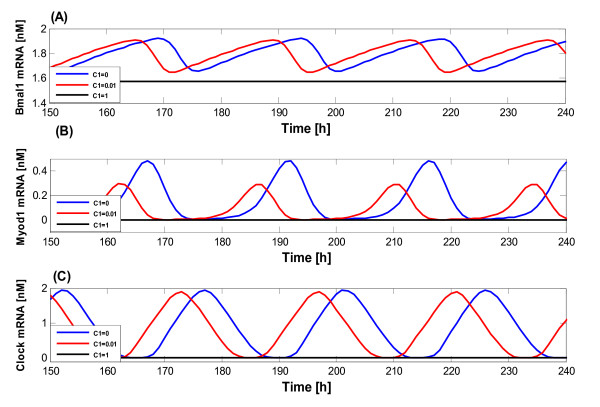
**Temporal behavior of the model with miRNA regulation**. Effect of the variation of the miR-206 production rate (*C*_1_) for the model. The blue, red and black curves indicate the cases for *C*_1 _= 0, 0.01 and 1 nM·h^-1^, respectively.

It is also interesting to explore the variation of the frequency and amplitude when *C*_1 _was set to 0.0, 0.01 and 1.0 nM·h^-1^, with all other rate constants kept fixed. The obtained results are shown in Figure 7, in which the blue curves represent the variation of Bmal1 mRNA, Myod1 mRNA and Clock mRNA in the absence of miRNA (as shown in Figures 7A, B and 7C, respectively). The red and black curves represent the conditions when *C*_1 _= 0.01 and 1.0 nM·h^-1 ^respectively. As seen from this figure, when *C*_1 _= 0.0 nM·h^-1^, the system still keeps relatively constant oscillations in the absence of miRNA, which might indicate that the regulation of miR-206 in the biological clock system is relatively weak [51]. But once the miR-206 is introduced (*C*_1 _= 0.01 nM·h^-1^), the oscillatory behavior of the system appears in an extremely constant manner. When *C*_1 _changes from 0.0 to 0.01 nM·h^1^, the amplitudes of oscillation for Bmal1 and Clock mRNAs do not show any significant changes, but the amplitude of oscillation for Myod1 mRNA decreases from 0.5 to 0.3 nM. The reason is that Myod1 directly activates the expression level of miR-206, thus resulting in the difference of expressions between Bmal1 and Myod1. All these results reveal that the sustained oscillations occur only within a certain range of parameter values. Beyond this range, the system tends to a steady state. So when more miRNA is produced (*C*_1 _= 1 nM·h^-1^) in the system, all the above three mRNAs evolve to a steady state and no oscillations occur. Clearly the over expression of miR-206 in the system disturbs the biological clock and makes the periodic oscillations collapsed. This result indicates that miRNAs are important in maintaining the tissue and cell normal function, and the abnormal expression of miRNAs may lead to unexpected diseases.

**Figure 8:**
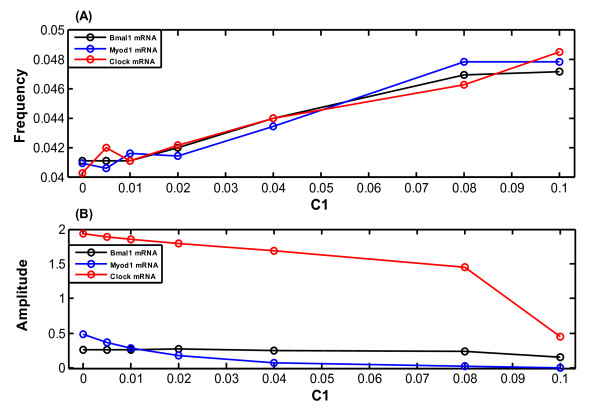
**Effect of the variation of the miR-206 production rate (*C*_1_, nM·h^-1^) on the model**. Variation of (A) the frequency and (B) the amplitude of oscillation in the model as a function of *C*_1 _(nM·h^-1^). Bmal1, Myod1 and Clock mRNAs are shown in black, blue and red, respectively.

In the next step, we carried out a detailed analysis to uncover how the system is affected by miR-206, when the amplitude and the frequency of oscillation for the above three representative components are calculated. Bmal1 mRNA, Myod1 mRNA and Clock mRNA are shown in black, blue and red in Figure 8, respectively. The results show that the frequencies of oscillation for all three mRNAs increase with the increase of *C*_1_, indicating that the temporal sequence of the gene production can be interfered (Figure 8A). The amplitudes of oscillation for Bmal1 and Myod1 mRNAs are almost constant and no significant changes are found though *C*_1 _has been changed from 0 to 0.1 nM·h^-1^. However, the Clock mRNA expression decreases by about 4 times (Figure 8B). This reduction in amplitude of the oscillation shows that the expression level of Clock mRNA can be affected when *C*_1 _increases. These results show that miR-206 may be a stabilizing factor on the frequency and amplitude of the oscillator system, thereby regulating the dynamics of the protein production and gene expression.

**Figure 9:**
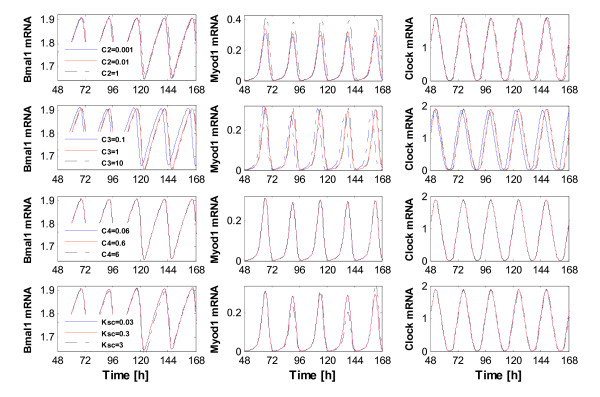
**Effect of the variation of parameters on the evolutions of Bmal1 mRNA, Myod1 mRNA and Clock mRNA**. The rate constants *C*_2_, *C*_3_, *C*_4 _and *K_sC _*are the degradation rate of miR-206, the formation rate of the *R_ISC _*complex, the maximum degradation rate of the *R_ISC _*complex and the synthesis rate of MYOD1, respectively.

In addition, the effects of the variation of parameters *C*_2 _(the degradation rate of the miR-206), *C*_3 _(the formation rate of the R*_ISC _*complex), *C*_4 _(the maximum degradation rate of the *R_ISC _*complex) and *K_sC _*(the synthesis rate of MYOD1) on the whole system are also investigated, respectively. Figure 9 shows the obtained results. As can be seen, when parameters *C*_2 _and *C*_3 _are increased by 10 folds compared to their normal values (Table 1), very small variations are observed for all three representative molecules, i.e., Bmal1 mRNA, Myod1 mRNA and Clock mRNA. Interestingly, the changes for other two parameters (*C*_4 _and *K_sC_*) also do not significantly affect the system, and the observed evolutionary curves with different parameter values are almost totally overlapped with each other. These results demonstrate that all the four parameters are not as significant as *C*_1_, the miRNA production rate, to the oscillator system, which is consistent with the findings obtained from the sensitivity analysis.

## Conclusion

MiRNAs play an important role in various biological functions and represent a potentially novel avenue by which biological timing processes can be regulated. In this paper, the dynamics of regulation by miR-206 were investigated based on the Clock-Myod1-miR-206 interacting positive and negative feedback loops controlling the timing of the circadian cycle in mammalian skeletal muscle. To this end, a system-theoretic approach by using Hill-type terms, Michaelis-Menten type and mass action kinetics is introduced in this work and the dynamics is implemented deterministically. The model outlined presently not only introduces miR-206 into the field of circadian timing, but also accurately predicts the clock periodicity and clock entrainment. A sustained periodic circadian oscillation occurs in the circadian clock system when the series of parameters were set with biologically reasonable values. In addition, this model reveals the possible existence of multiple sources of biological oscillatory behavior, and provides a plausible mechanism through which tissue-specific factors such as Myod1 and miR-206 can convey unique tissue requirements to the circadian clock. In addition, the results point out the important effects of parameter variations such as the miR-206 synthesis on the regulation of the mammalian clock system, which not only changes the amplitude but also alters the frequency of the oscillations of the deterministic system. In other words, miR-206 plays a significant role on the dynamics of regulatory systems, both by affecting the level of the gene expression and by interfering with the temporal sequence of the gene production or transmission.

In short, the essential qualitative features of these results indicate that the miRNA regulation may be one of the primary means for controlling the period of oscillatory molecules and biochemical pathways within mammalian circadian clock. We provide testable hypothesis for experimental biologists to further investigate miRNA's functional roles in regulating cellular processes and development. The challenge for future studies will be a focus on the modulation of the miRNA expression as a potentially powerful diagnostic and therapeutic approach to treat cardiac and skeletal muscle disease.

## Methods

### Model description

**Figure 10:**
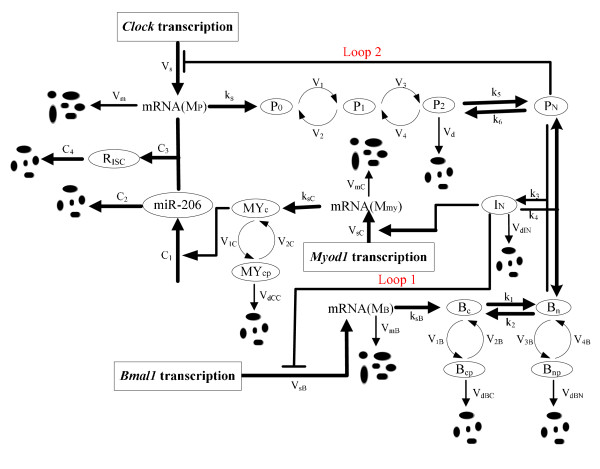
**A Clock-Myod1-miR-206 feedback loop controlling the timing of the circadian cycle in skeletal muscle**. The subscripts *c*, *n*, *cp *and *np *of the symbols represent for cytosolic, nuclear, cytosolic phosphorylated and nuclear phosphorylated, respectively.

In this work, a model of the mammalian circadian core oscillator which is mediated by miRNA was developed, using a series of ordinary differential equations (ODEs) (Figure 10). The focus is put specifically on the essential structure of the molecular network to characterize the role of the positive and negative feedback loops regulated by miR-206.

Evidence to support the model is as follows:

1) In the cell nucleus, the genes of Myod1 and Bmal1 are transcribed into the corresponding Myod1 and Bmal1 mRNAs (denoted *M_my _*and *M_B_*). Then these mRNAs are transported to the cytoplasm and degraded.

2) In the cytoplasm, the two mRNAs are translated into unphosphorylated proteins, i.e., the MYOD1 and BMAL1 (denoted by *MY_C _*and *B_c_*).

3) These unphosphorylated proteins further undergo reversible phosphorylation (with phosphorylated forms denoted by *MY_cp _*and *B_cp_*) and degradation processes.

4) The cytosolic BMAL1 protein is reversibly transported into the nucleus. In turn, the nuclear BMAL1 protein proceeds reversible phosphorylation and then degrades in nucleus (with unphosphorylated and phosphorylated BMAL1 denoted by *B_n _*and *B_np_*, respectively).

5) The model also incorporates the Clock gene transcription, i.e., the Clock mRNA (*M_P_*), which transports into the cytoplasm where it is then translated into the CLOCK protein (*P*_0_) and degrades. In the cytoplasm, the CLOCK protein can be reversibly phosphorylated from the form of *P*_0 _into *P*_1 _and *P*_2_, successively.

6) The latter form *P*_2 _is degraded and transported into the nucleus (*P_N_*), and the nuclear form of protein *P_N _*subsequently represses its gene expression and produces a negative feedback control loop (Figure 10, Loop 2) [24]. Because the expression of Clock is considered to be constitutive and give rise to a high, constant level of cytosolic and nuclear CLOCK proteins [52], we assume that once entering the nucleus, the unphosphorylated BMAL1 immediately forms a complex with the Clock [40] (the complex is denoted by *I_N_*).

7) In the nucleus, the BMAL1-CLOCK heterodimer increases the rate of transcription of the corresponding gene, Myod1 [53]. At the same time, it represses the transcription of Bmal1 gene through exerting a negative autoregulation on the pathway (Figure 10, Loop 1) [54], and this regulatory effect of BMAL1 is described as a direct and negative autoregulation type.

8) In turn, the Myod1 gene, which is known to regulate the miR-206 expression [23] has been shown to drive the expression of the primary miR-206 transcript AK132542, leading to increased expression of miR-206 [22]. And miR-206 subsequently represses the expression of Clock by binding to the Clock mRNA and forms the complex RNA-induced silence complex (*R_ISC_*) and then degrades [32].

In summary, this model displays the essential components and characteristics of the circadian clock, and provides a plausible mechanism through which the tissue-specific factors such as Myod1 and miR-206 can convey unique tissue requirements to the circadian clock. The detailed pathway is shown in Figure 10.

### Rate equations

Deterministic models based on ODEs can provide insights into the understanding of the biological pathway, as how the overall behaviors depend on a set of constituent reactions. The whole circadian clock pathway is modeled using a set of ODEs which were numerically solved by stiff method. The variables of the system represent the concentrations of each species like mRNAs or proteins (Figure 10).

In this paper, we develop a detailed distinctly dynamic model of the mammalian circadian clock mediated by miR-206 by using Hill-type terms, Michaelis-Menten type and mass action kinetics, which are the mathematical models that explain and predict the behaviors of the molecules in dynamic equilibrium. The time evolution of the model is governed by a system of 19 kinetic Equations (1)-(19). The ODEs of the dynamic model are expressed as follows:

(a) genes of Bmal1, Clock and Myod1:

(1) $$\frac{d\left(\left[Bmal1\right]\right)}{dt}=d_{1}-d_{2}\;\left[Bmal\;1\right]\;-V_{sB}\frac{{K_{IB}}^{m_{1}}}{{K_{IB}}^{m_{1}}+{\left[I_{N}\right]}^{m_{1}}}$$

(2) $$\frac{d\left(\left[Clock\right]\right)}{dt}=d_{5}-d_{6}\left[Clock\right]-V_{s}\frac{{K_{I}}^{m_{2}}}{{K_{I}}^{m_{2}}+{\left[P_{N}\right]}^{m_{2}}}$$

Although Eqs. (1) and (2) are applied for different genes, the underlying principles and formulations are similar: the first two terms represent the generation and degradation of given genes, while the last one represents the inhibition of gene transcription by the negative feedback control [35,55].

(3) $$\frac{d\left(\left[Myod1\right]\right)}{dt}=d_{3}-d_{4}\left[Myod1\right]-V_{sC}\frac{{\left[I_{N}\right]}^{n_{1}}}{{K_{AC}}^{n_{1}}+{\left[I_{N}\right]}^{n_{1}}}$$

In Eq. (3), the first two terms are the same to Eqs. (1) and (2). The last term represents the activation of the gene transcription by the complex *I_N _*[52].

(b) mRNAs of Bmal1, Myod1 and Clock:

(4) $$\begin{array}{cc} \frac{d\left(\left[M_{B}\right]\right)}{dt}= & V_{sB}\frac{{K_{IB}}^{m_{1}}}{{K_{IB}}^{m_{1}}+{\left[I_{N}\right]}^{m_{1}}}-V_{mB}\frac{\left[M_{B}\right]}{K_{mB}+\left[M_{B}\right]}\\ & -k_{dmd}\left[M_{B}\right] \end{array}$$

(5) $$\begin{array}{cc} \frac{d\left(\left[M_{my}\right]\right)}{dt}= & V_{sC}\frac{{\left[I_{N}\right]}^{n_{1}}}{{K_{AC}}^{n_{1}}+{\left[I_{N}\right]}^{n_{1}}}-V_{mC}\frac{\left[M_{my}\right]}{K_{mC}+\left[M_{my}\right]}\\ & -k_{dmc}\left[M_{my}\right] \end{array}$$

We define *M_B _*and *M_my _*as the concentrations of mRNA product at time *t*, and then the variations of the two mRNAs with time are summarized by Eqs. (4) and (5). The two equations describe the synthesis, maximum degradation and nonspecific degradation of the Bmal1 mRNA and Myod1 mRNA, respectively.

(6) $$\begin{array}{c} \frac{d\left(\left[M_{P}\right]\right)}{dt}=V_{s}\frac{{K_{I}}^{m_{2}}}{K_{I}^{m_{2}}+{\left[P_{N}\right]}^{m_{2}}}-V_{m}\frac{\left[M_{P}\right]}{K_{m}+\left[M_{P}\right]}\\ \;-C_{3}\;\left[miR\text{ - }206\right]\;\left[M_{P}\right] \end{array}$$

The first two terms in Eq. (6) are similar to Eq. (5) by representing the production and degradation of *M_P _*(the mRNA of Clock). The third term represents the formation of the miR-206-*M_P _*(*R_ISC_*) complex.

(c) reversibly phosphorylated and non-phosphorylated proteins MYOD1 in the cytoplasm:

(7) $$\begin{array}{c} \frac{d\left(\left[MY_{C}\right]\right)}{dt}=-V_{1C}\frac{\left[MY_{C}\right]}{K_{p3}+\left[MY_{C}\right]}+V_{2C}\frac{\left[MY_{CP}\right]}{k_{dp3}+\left[MY_{CP}\right]}\\ \;-k_{dnc}\left[MY_{C}\right]+k_{sC}\left[M_{my}\right] \end{array}$$

(8) $$\begin{array}{c} \frac{d\left(\left[MY_{cp}\right]\right)}{dt}=V_{1C}\frac{\left[MY_{c}\right]}{K_{p3}+\left[MY_{c}\right]}-V_{2C}\frac{\left[MY_{cp}\right]}{K_{dp3}+\left[MY_{cp}\right]}\\ \;\;\;-k_{dn4}\left[MY_{cp}\right]-V_{dCC}\frac{\left[MY_{cp}\right]}{k_{d3}+\left[MY_{cp}\right]} \end{array}$$

where the phosphorylation and dephosphorylation in the first two terms of each equation are governed by the Michaelis-Menten kinetics. The third term in above two equations represents the nonspecific degradation of the non-phosphorylated and phosphorylated cytosolic MYOD1s, respectively. The last term in Eq. (7) represents the synthesis of the cytosolic MYOD1, while in Eq. (8) represents the maximum degradation of the phosphorylated cytosolic MYOD1 using the Michaelis-Menten kinetics, respectively.

(d) phosphorylated and non-phosphorylated proteins BMAL1 and CLOCK in the cytoplasm and nucleus:

(9) $$\begin{array}{c} \frac{d\left(\left[B_{n}\right]\right)}{dt}=-V_{3B}\frac{\left[B_{n}\right]}{K_{p2}+\left[B_{n}\right]}+V_{4B}\frac{\left[B_{np}\right]}{K_{dp2}+\left[B_{np}\right]}\\ \;\;\;\;\;+k_{1}\left[B_{c}\right]+k_{4}\left[I_{N}\right]-k_{3}\left[P_{N}\right]\left[B_{n}\right]\\ \;\;\;\;\;-k_{2}\left[B_{n}\right]-k_{dn3}\left[B_{n}\right] \end{array}$$

(10) $$\begin{array}{c} \frac{d\left(\left[Bc\right]\right)}{dt}=-V_{1B}\frac{\left[B_{c}\right]}{K_{p1}+\left[B_{c}\right]}+V_{2B}\frac{\left[B_{cp}\right]}{K_{dp1}+\left[B_{cp}\right]}\\ \;\;\;\;\;+k_{sB}\left[M_{B}\right]+k_{2}\left[B_{n}\right]\\ \;\;\;\;\;-k_{1}\left[B_{c}\right]-k_{dn1}\left[B_{c}\right] \end{array}$$

These two equations describe the synthesis and degradation of the cytosolic and nuclear BMAL1 proteins, respectively. The first two terms are similar to Eqs. (7) and (8), while the remaining terms correspond to the synthesis or degradation of the proteins by using the mass action rules.

(11) $$\begin{array}{c} \frac{d\left(\left[B_{cp}\right]\right)}{dt}=V_{1B}\frac{\left[B_{c}\right]}{K_{p1}+\left[B_{c}\right]}-V_{2B}\frac{\left[B_{cp}\right]}{K_{dp1}+\left[B_{cp}\right]}\\ \;\;\;-k_{dn2}\left[B_{cp}\right]-V_{dBC}\frac{\left[B_{cp}\right]}{K_{d1}+\left[B_{cp}\right]} \end{array}$$

(12) $$\begin{array}{c} \frac{d\left(\left[B_{np}\right]\right)}{dt}=V_{3B}\frac{\left[B_{n}\right]}{K_{p2}+\left[B_{n}\right]}-V_{4B}\frac{\left[B_{np}\right]}{K_{dP2}+\left[B_{np}\right]}\\ \;\;\;-k_{dn6}\left[B_{np}\right]-V_{dBN}\frac{\left[B_{np}\right]}{K_{d2}+\left[B_{np}\right]} \end{array}$$

Eqs. (11) and (12) describe the synthesis and degradation of the phosphorylated cytosolic or nucleus BMAL1 proteins, respectively. The principles for the two equations are the same as Eq. (8).

(13) $$\frac{d\left(\left[P_{0}\right]\right)}{dt}=k_{s}\left[M_{P}\right]-V_{1}\frac{\left[P_{0}\right]}{K_{1}+\left[P_{0}\right]}+V_{2}\frac{\left[P_{1}\right]}{K_{2}+\left[P_{1}\right]}$$

(14) $$\begin{array}{c} \frac{d\left(\left[P_{1}\right]\right)}{dt}=V_{1}\frac{\left[P_{0}\right]}{K_{2}+\left[P_{0}\right]}-V_{2}\frac{\left[P_{1}\right]}{K_{2}+\left[P_{1}\right]}\\ \;\;\;\;\;-V_{3}\frac{\left[P_{1}\right]}{K_{3}+\left[P_{1}\right]}+V_{4}\frac{\left[P_{2}\right]}{K_{4}+\left[P_{2}\right]} \end{array}$$

(15) $$\begin{array}{c} \frac{d\left(\left[P_{2}\right]\right)}{dt}=-V_{d}\frac{\left[P_{2}\right]}{K_{vd}+\left[P_{2}\right]}+V_{3}\frac{\left[P_{1}\right]}{K_{3}+\left[P_{1}\right]}\\ \;\;\;\;\;-V_{4}\frac{\left[P_{2}\right]}{K_{4}+\left[P_{2}\right]}+k_{6}\left[P_{N}\right]-k_{5}\left[P_{2}\right] \end{array}$$

(16) $$\begin{array}{c} \frac{d\left(\left[P_{N}\right]\right)}{dt}=-k_{6}\left[P_{N}\right]+k_{5}\left[P_{2}\right]\\ \;\;\;\;\;+k_{4}\left[I_{N}\right]-k_{3}\left[P_{N}\right]\left[B_{n}\right] \end{array}$$

Equations (13)-(15) relate to the synthesis and degradation of phosphorylated and non-phosphorylated cytosolic CLOCK proteins. Eq. (16) refers to the variation of the concentration of nucleus CLOCK protein with time. The principles are similar to the above equations.

(e) BMAL1- CLOCK complex in nucleus:

(17) $$\begin{array}{c} \frac{d\left(\left[I_{N}\right]\right)}{dt}=k_{3}\left[P_{N}\right]\left[B_{n}\right]-k_{4}\left[I_{N}\right]\\ \;\;\;\;\;-V_{dIN}\frac{\left[I_{N}\right]}{K_{d4}+\left[I_{N}\right]}-k_{dn5}\left[I_{N}\right] \end{array}$$

where the first term corresponds to the synthesis of complex *I_N_*, while the last three terms represent the dissociation, maximum degradation and nonspecific degradation of *I_N_*, respectively.

(f) miR-206 and *R_ISC_*:

(18) $$\begin{array}{c} \frac{d\left(\left[miR\text{ - }206\right]\right)}{dt}=\left(V_{sM}\frac{{\left[MY_{C}\right]}^{n_{2}}}{{K_{AM}}^{n_{2}}+{\left[MY_{C}\right]}^{n_{2}}}+C_{1}\right)\\ \;\;\;\;\;\;\;\;-C_{2}\left[miR\text{ - }206\right]-C_{3}\left[miR\text{ - }206\right]\left[M_{P}\right] \end{array}$$

(19) $$\frac{d\left(\left[R_{ISC}\right]\right)}{dt}=C_{3}\left[miR\text{ - }206\right]\left[M_{P}\right]-C_{4}\left[R_{ISC}\right]$$

The concentration change of miR-206 is given by Eq. (18) and that for *R_ISC _*is given by Eq. (19). These two equations are similar in that they describe the synthesis and degradation of miR-206 and *R_ISC _*respectively, but different in that the synthesis of miR-206 is described by the Hill-type function.

The definition of all the parameters in Eqs. (1)-(19) is shown in Table 1. The concentrations of all species are defined with respect to the total cell volume, which is denoted by the subscripts of *c*, *n*, *cp *or *np *for the cytosolic, nuclear, cytosolic phosphorylated or nuclear phosphorylated terms, respectively.

### Dynamic sensitivity analysis

The definition of sensitivity analysis (*S_A_*) is that *S_A _*studies the relationships between the information flowing in and out of a model. It is a step in the modeling process aiming at identifying the important uncertainties for the purpose of prioritizing the additional data collection or research. It is used to determine how "sensitive" a model is to those changes occurring in the parameter values or in the structure of the model. A dynamic sensitivity value reflects a relative relationship between the magnitudes of a parameter (input) and a state variable (output) at any time. Thus, the measure of the sensitivity is that the sensitivity of an item is taken to be the output with respect to a single parameter *X_i_*:

(20) $$S_{A}=\frac{\partial Y}{\partial X_{\text{i}}}$$

The quantity *S_A _*is the local sensitivity index of the state variable *Y *(output) relative to the parameter *X_i_*, and the sensitivity of each variable corresponds to a specified parameter. In order to obtain the scaled sensitivity coefficient *S_A _*that is dimensionless and plotted for maintaining the visual presentation drawing, the results are normalized.

## Competing interests

The authors declare that they have no competing interests.

## Authors' contributions

Conceived and designed the experiments: YW, YL. Performed the experiments: WZ. Analyzed the data: WZ, XW, LW. Wrote the paper: WZ, YL. All authors have read and approved the final manuscript.
